# Forgetting Unwanted Memories: Active Forgetting and Implications for the Development of Psychological Disorders

**DOI:** 10.3390/jpm11040241

**Published:** 2021-03-26

**Authors:** Marco Costanzi, Beatrice Cianfanelli, Alessandro Santirocchi, Stefano Lasaponara, Pietro Spataro, Clelia Rossi-Arnaud, Vincenzo Cestari

**Affiliations:** 1 Department of Human Sciences, Lumsa University, 00193 Rome, Italy; b.cianfanelli@lumsa.it (B.C.); s.lasaponara@lumsa.it (S.L.); 2 Department of Psychology, Sapienza University, 00185 Rome, Italy; alessandro.santirocchi@uniroma1.it (A.S.); clelia.rossi-arnaud@uniroma1.it (C.R.-A.); vincenzo.cestari@uniroma1.it (V.C.); 3 Department of Economy, Universitas Mercatorum, 00100 Rome, Italy; pietro.spataro@unimercatorum.it

**Keywords:** forgetting, neurotransmitter system, psychopathologies

## Abstract

Intrusive memories are a common feature of many psychopathologies, and suppression-induced forgetting of unwanted memories appears as a critical ability to preserve mental health. In recent years, biological and cognitive studies converged in revealing that forgetting is due to active processes. Recent neurobiological studies provide evidence on the active role of main neurotransmitter systems in forgetting, suggesting that the brain actively works to suppress retrieval of unwanted memories. On the cognitive side, there is evidence that voluntary and involuntary processes (here termed “intentional” and “incidental” forgetting, respectively) contribute to active forgetting. In intentional forgetting, an inhibitory control mechanism suppresses awareness of unwanted memories at encoding or retrieval. In incidental forgetting, retrieval practice of some memories involuntarily suppresses the retrieval of other related memories. In this review we describe recent findings on deficits in active forgetting observed in psychopathologies, like post-traumatic stress disorder, depression, schizophrenia, and obsessive-compulsive disorder. Moreover, we report studies in which the role of neurotransmitter systems, known to be involved in the pathogenesis of mental disorders, has been investigated in active forgetting paradigms. The possibility that biological and cognitive mechanisms of active forgetting could be considered as hallmarks of the early onset of psychopathologies is also discussed.

## 1. Introduction

There is increasing empirical research suggesting that intrusive memories are a common feature of many mental disorders [1,2,3,4,5,6]. In patients, intrusions are due to the involuntary retrieval of unwanted memories [7,8,9], which tend to be repetitive, uncontrollable, and distressing [1]. Suppression of unwanted memories appears to be a critical ability to avoid their unintended influence, thus preserving mental health [9,10]. In healthy subjects, the ability to intentionally suppress memory retrieval (a phenomenon known as suppression-induced forgetting) has been associated with a lesser presence of distressing, intrusive memories for a traumatic movie [11]. On the contrary, impaired suppression-induced forgetting has been associated with worse mental health and has been found in individuals suffering from post-traumatic stress disorder [12], rumination [13,14], anxiety [15,16], and depression [17,18,19,20].

Forgetting is the inability to recall previously consolidated memories. Natural time-dependent decay of memory traces, change of context between acquisition and retrieval, and interference have all been considered mechanisms responsible for the inability to recall memories. Aside from the aforementioned passive mechanisms, a number of more recent studies indicate that forgetting is also due to active processes, which actively work to eliminate memories from the brain [21,22,23,24].

On the cognitive side, two main mechanisms for active forgetting have been proposed: incidental and intentional forgetting [22,25,26]. Here, we use the terms “incidental” and “intentional” slightly differently from the way they have been typically adopted in memory studies. In the latter, incidental instructions refer to situations in which participants are not explicitly told to memorize to-be-encoded items and are unaware of the impeding memory test. Intentional instructions refer to situations in which participants are explicitly told to remember the presented items in view of a later memory task [27]. That is, in these studies the two terms are used to indicate the degree of intentionality of the encoding processes. In contrast, in the present reviews the same terms are used to reflect the degree of intentionality of the forgetting processes: along this line, incidental forgetting refers to situations in which participants are not explicitly instructed to forget, whereas intentional forgetting refers to situations in which participants are asked to deliberately forget some previously learned information [26].

**Figure 1:**
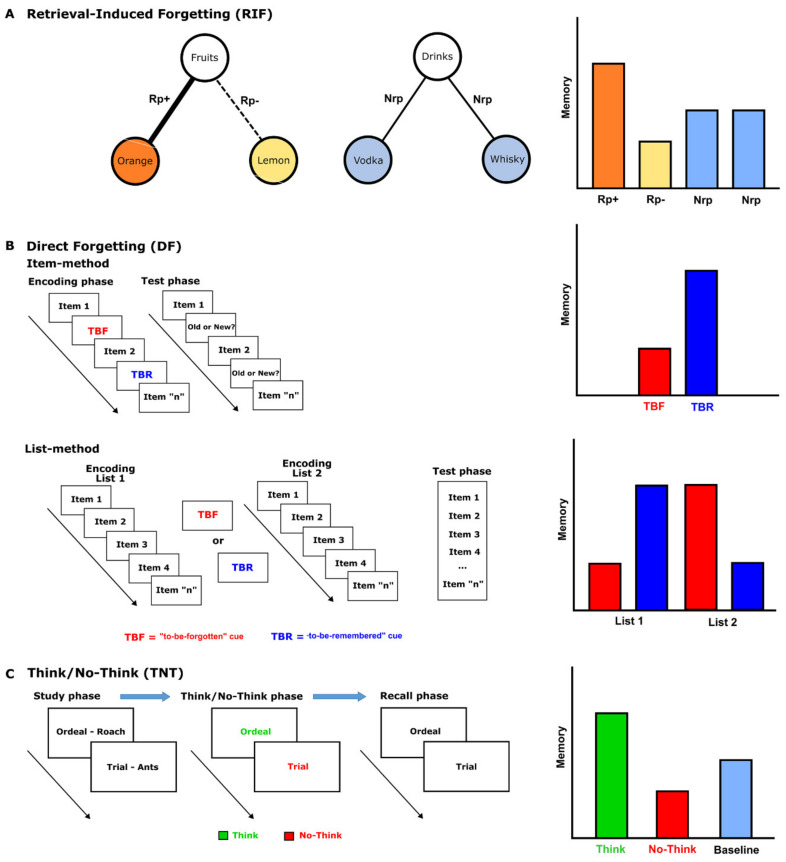
Schematic representations and procedural overview along with the typical pattern of findings of (**A**) the semantic retrieval-induced forgetting (RIF) paradigm, (**B**) item and list-methods for studying directed forgetting (DF), and (**C**) the think/no-think task (TNT).

More specifically, incidental forgetting occurs when retrieval of some memories involuntarily suppresses the retrieval of other, related, memories (retrieval-induced forgetting [21]) or when, according to a number of authors, memories that threaten our positive self-image are involuntarily repressed (e.g., Freudian repression). Although repression is at the heart of a heated debate [28], it has been described as occurring when a thought or a memory is too painful for an individual, so the person unconsciously pushes the information out of consciousness and becomes unaware of its existence [29]. Retrieval-induced forgetting (RIF), on the other hand, occurs when retrieval practice of items belonging to a category causes forgetting of unpracticed items belonging to the same category, in the absence of any instruction to voluntarily forget these items [30,31,32,33]. Such RIF studies typically involve three stages: study, retrieval practice, and final test [34]. During the study phase, participants are presented with a series of category–exemplar pairs (e.g., fruits–orange, drinks–vodka), under the instructions of studying them for a subsequent test or simply thinking about the associations. In the retrieval practice phase, participants are asked to retrieve half of the exemplars from half of the categories by completing category-plus-item-specific cues (e.g., fruit: or___). Often, participants undergo several rounds of retrieval practice before beginning the final phase, in which their ability to retrieve the exemplars is tested. Three types of exemplars are examined in the final recall task: Rp+ items refer to practiced exemplars (orange); Rp− items refer to non-practiced items from practiced categories (lemon); and Nrp items refer to exemplars from non-practiced categories (vodka). Two results classically emerge when using this paradigm: first, Rp+ items are recalled better than Rp− and Nrp items; second, Rp− items are recalled less well than Nrp items. It is exactly the latter finding which is usually referred to as RIF (see Figure 1A). Although several mechanisms have been proposed to account for this phenomenon, most of them can be grouped into the broad category of inhibition-based forgetting theories [34]. According to this perspective, attempts to retrieve practiced exemplars from memory cause associated items to become activated. Since this activation creates competition, non-practiced items from the same category are inhibited in order to selectively retrieve the target items. In this way, inhibition has the key function to reduce interference from non-practiced items during retrieval practice. However, in the final memory task, this same inhibition leads to poorer recall of Rp− items [30,31]. So, for example, when orange is retrieved during retrieval practice, the associated item, lemon, may become incidentally activated; to facilitate the retrieval of orange, lemon is inhibited, thus rendering it less accessible on the final test.

Intentional forgetting, on the other hand, occurs when cognitive mechanisms are voluntarily engaged to weaken memories. Directed-forgetting (DF) and think/no-think (TNT) are experimental paradigms used to investigate intentional forgetting. In the former, two procedures have been used, depending on whether the instructions to remember or to forget are directed towards single items (item-method) or a list of items (list-method) [35]. In the item-method DF, participants are presented with individual items during the encoding phase, and each item is followed by either a “remember” or a “forget” cue. The common finding is that, in a later recall task, items followed by the instruction “to-be-forgotten” are worse remembered than those followed by the instruction “to-be-remembered”. In the list-method DF, participants are instead presented with two different lists of items during the encoding phase: the first list is followed by a forget or remember cue, whereas the second list is usually followed by a remember cue. In this case, two different effects emerge in the final memory task: the forgetting of list-1 items (i.e., impaired recall for the first list of items when subjects are instructed to forget this list, relative to when they are instructed to remember it) and the enhancement of list-2 items (i.e., improved recall for the second list of words when subjects are instructed to forget the first list, relative to when they are instructed to remember the first list; Figure 1B) [36]. One of the earliest theories that was proposed to account for DF is the selective rehearsal hypothesis [37]. Put simply, it states that presentation of the forget cue leads participants to stop the rehearsal of to-be-forgotten items and devote all their resources to the processing of to-be-remembered items, thus producing the memory enhancement [38]. An alternative hypothesis proposes that the benefit in the recall of list-2 might be explained by differences in the type of strategies used to encode the two lists (i.e., participants instructed to forget list-1 items typically use a deeper strategy to encode list-2 items). There is indeed evidence showing that modulating the encoding strategies in the list-method DF (e.g., using a shallow or a deep strategy to encode both lists) abolishes the benefits in remembering the list-2 but do not alter the inhibition of list-1 [39]. These results suggest that participants treat the “to-be-forgotten” and “to-be-remembered” lists as separate events and do not maintain the context in which they initially encoded list-1 when they are encoding list-2 [39,40]. Lastly, the third hypothesis is provided by the active inhibition account. According to this view, when participants are presented with the forget cue after the first list, they initiate an inhibitory process that suppresses activation of that list, so as to facilitate the learning of the subsequent list. The result is that memory of the first list suffers from inhibition, whereas memory of the second list benefits because the first list can no longer cause proactive interference [35,37]. In this respect, Anderson and Hanslmayr (2014) argued that the list- and item-methods differ in the target of forgetting: the item method leads to the inhibition of individual items, while the list method typically directs people to inhibit a set of items defined by temporal context (i.e., “the previous list”). Hence, in both item- and list-methods the encoding seems to be disrupted by an active inhibitory control mechanism that limits long-term memory formation for to-be-forgotten items or lists, respectively [35]. This interpretation has been recently supported by neurophysiological evidence showing that memory suppression in both item- and list-method DF is mediated by the inhibitory activity of prefrontal cortex on the medial temporal lobe [33]. Specifically, studies using connectivity analyses showed that increased activity in the right dorsolateral prefrontal cortex during forget trials predicted decreased activity in the left hippocampus, especially during successful intentional forgetting [41]. In another study, it was found that stimulating the dorsolateral prefrontal cortex with repetitive transcranial magnetic stimulation during a forget instruction increased the magnitude of the DF effect in the list-method [42].

Lastly, in the TNT paradigm, forgetting is obtained by asking subjects to suppress thoughts about stimuli cued by the instruction “no-think” [35]. In this paradigm, participants study cue–target pairs (i.e., word pairs such as ordeal–roach) and are then repeatedly trained to recall the second terms (the targets: roach) in response to the first terms (the cues: ordeal). Then, in the TNT phase, the cues are re-presented together with think/no-think instructions: when the cues appear with the “think” signal, participants have to recall the targets (“think” items); in contrast, when the cues appear with the “no-think” signal, they must avoid recalling the targets (“no-think” items). To measure the effectiveness of the think/no-think instructions, participants receive a final test in which they are given each cue and are asked to recall the associated target. Here, the typical finding is that “no-think” items are worse remembered than “think” items (Figure 1C). One important difference between the TNT and DF paradigms is that only the former assesses the ability to inhibit information that has been well learned [43]. In the DF procedures, participants study test items on an item-by-item or list-by-list basis, making it difficult to determine whether forgetting is due to inhibition or to simply not encoding the information into memory. In contrast, in the TNT paradigms participants are first required to learn the studied items until being able to retrieve half or two-thirds of them. This methodology ensures that participants have successfully encoded the items that are then asked to reinforce (“think” trials) or inhibit (“no-think” trials). The difference between the two paradigms implies that inhibitory control acts at different levels of the memory processing: in the DF paradigm memory recall is impaired by inhibition at encoding, while in TNT paradigm memories are suppressed by inhibition at retrieval [35]. More specifically, the participants’ task in the TNT paradigm is to prevent encoded information from coming to mind; to this purpose, an active mechanism must stop the retrieved items from reaching consciousness (Murray et al., 2011). According to Anderson and Hanslmayr [35] one such mechanism is inhibition. That is, when participants are instructed to “not think” about a learned word, they must actively inhibit the desire to think about, or recall, that particular word. In agreement with this proposal and similar to DF, retrieval suppression in TNT paradigm appears to be achieved by inhibitory control mechanisms mediated by the prefrontal cortex [35]. In particular, Anderson et al. (2004) found that “no-think” trials were accompanied by increased activation of bilateral dorsolateral and ventrolateral prefrontal cortex, as well as by reduced activity bilaterally in the hippocampus.

Both incidental and intentional forgetting are involved in emotional memory control [10,44,45], and deficits in regulating such memories are known to play a key role in the onset of psychopathological disorders [45,46]. In fact, recent neurobiological studies seem to confirm that the brain has the capacity to actively erase memories through the actions of molecular cascades involved in several neuronal functions [22,47]. In particular, dopamine-related intracellular cascades, receptor trafficking, spine shrinkage involving NMDA-dependent long-term depotentiation, and adult neurogenesis remodeling of hippocampal circuits emerge as biological mechanisms actively involved in memory forgetting [22,47].

The discovery of neurobiological pathways actively involved in forgetting could be relevant in investigating possible overlapping mechanisms between deficits in suppression of unwanted memories and the onset of psychopathological disorders [35,48,49].

Changes in neurotransmission have been shown to alter behaviors and to play a pivotal role in the onset of psychopathological disorders. Among neurotransmitters and hormones, the dopaminergic system seems to be relevant for the onset of anxiety disorders, schizophrenia, and pathological gambling, as well as for mood swings; the noradrenergic system seems to be involved in the occurrence of attentional deficit hyperactive disorder (ADHD), depression and anxiety disorders; the cholinergic system is involved in Alzheimer disease (AD), ADHD, chronic fatigue, and depression; the serotoninergic system is involved in depression, impulse control disorders, obsessive-compulsive disorder (OCD), and suicidal behavior; the glutamatergic system has been implicated in the development of schizophrenia and OCD; the GABAergic system is involved in anxiety disorders; and glucocorticoids have been implicated in stress-induced pathologies, like post-traumatic stress disorder (PTSD) [50,51,52,53,54,55]. Interestingly, such neurotransmitters and hormones are involved in memory formation and forgetting [56,57,58,59,60,61].

In the first part of the present article, we review findings on active forgetting in psychological disorders, especially on its possible role as a potential cognitive marker for the early identification of psychopathologies. In the second part, we review literature on the role of the main neurotransmitter and hormone systems in forgetting.

## 2. Intentional and Incidental Forgetting in Post-Traumatic Stress Disorder

Post-traumatic stress disorder (PTSD) occurs when people are exposed to a horrific traumatic event, which involves threatened death, actual or threatened serious injury, or actual or threatened sexual violation [62]. PTSD patients usually show an impairment in the voluntary retrieval of autobiographical memory linked to the traumatic experience, an increased incidence of involuntary memories (i.e., flashback), as well as overgeneralization and avoidance of contexts resembling trauma. Not all people exposed to similar traumatic events develop PTSD. Many of them exhibit time-limited distress, and intrusions decline naturally over the first few months after trauma [63]. However, traumatic memories can be unforgettable and interfere with normal life also in the absence of PTSD. Recently, Millon and colleagues (2018) found that women who experienced sexual violence in adolescence, but who did not develop PTSD, reported particularly strong memories for stressful life events in adulthood. In these women, intrusive memories of autobiographical stressful events correlated with the presence of altered cognitions related to traumatic life experiences and with an increased level of anxiety and depression [64].

The diversity of responses to the traumatic event suggests that differences in how people process traumatic experiences in the memory system could play a causal role in the development and maintenance of PTSD [65,66,67]. Large individual differences in the ability to suppress unwanted memories have been observed in healthy subjects [68]. Furthermore, experimental findings on the inability of PTSD patients to inhibit trauma-related thoughts suggest that difficulties in memory forgetting due to inhibitory control deficits could be involved in the onset of PTSD [15,17,66,68,69,70,71]. In the past twenty years, a number of studies investigating intentional forgetting in PTSD patients have been carried out using both the DF and TNT paradigms [11,12,67,72,73,74,75,76,77,78,79,80,81]. In comparison with healthy controls and traumatized patients who have not developed a PTSD, PTSD patients tested with the DF paradigms showed memory deficits for “to-be-remembered” neutral and emotional words, but not for trauma-related ones, indicating a reduced ability to memorize information [67,73,75,77]. Despite this general memory impairment, the forgetting rate of PTSD patients is similar to that of healthy controls, especially when trauma-related memories are considered [67,73,75,76,78,81]. These results indicate that PTSD patients have a preserved DF effect, suggesting that the ability to inhibit memory during the encoding is spared in PTSD.

However, although the DF effect seems to be largely preserved in PTSD patients, there is evidence showing a reduced DF effect in specific conditions in which the vulnerability to dissociate as a consequence of traumatic stressors in these patients is taken into account. In PTSD, dissociation is considered as an alteration of memory functioning due to the inability to disengage attention from threatening stimuli.

Zoellner and collaborators (2003) found that PTSD patients submitted to a dissociative-inducing procedure (based on the administration of Velten-like phrases derived from the Peritraumatic Dissociative Experiences Questionnaire) did not show the DF effect [81]. Similarly, healthy subjects at risk for developing a PTSD, who reported high score in the Dissociative Experiences Scale, displayed a reduction in the DF effect for trauma-related words when a concurrent secondary task (dived-attention condition) was performed during the primary DF task [76].

Furthermore, EEG studies in adults with a history of childhood abuse and high scores in the Post-traumatic Stress Diagnostic Scale revealed an increase in the alpha coherence of frontoparietal networks—known to be involved in the allocation of attention during working memory tasks [82]—after word presentation in a DF task. Such an increase in alpha coherence was higher in participants with earlier traumas [83].

On the basis of results obtained in PTSD patients submitted to the DF paradigm, it is possible to envisage that differences in the ability to allocate attentional resources could affect memory inhibition, predicting the onset of PTSD after trauma exposure.

As concerns the suppression of memory retrieval assessed through the TNT paradigm, PTSD patients showed an impairment in the ability to suppress memory retrieval for both emotional and neutral pictures [12,77,79]. The deficit in suppressing memories positively correlated with symptom severity: the higher the score in the Post-traumatic Stress Diagnostic Scale, the higher the recognition of items associated to the no-think instruction [12,79].

Interestingly, a magnetoencephalography analysis in PTSD patients revealed that the deficit in suppression-induced forgetting in the TNT task was associated with an increased gamma power—a neural marker of sensory long-term memory traces—recorded during the presentation of no-think items, suggesting that PTSD patients experienced a rebound of sensory memory representations when trying to stop unwanted memories [79].

Regarding incidental forgetting, Amir and collaborators (2009) found that traumatized subjects with or without PTSD submitted to a retrieval practice paradigm for neutral, negative, and positive words showed a slight reduction in the typical RIF effect observed in non-traumatized control subjects. Moreover, PTSD patients remembered fewer practiced words than traumatized subjects without PTSD and healthy non-traumatized controls [84].

Taken together, the results on active forgetting in PTSD suggest that a deficit in the ability to intentionally suppress the retrieval of unwanted memories could be a cognitive hallmark for the risk of developing PTSD after a trauma.

## 3. Intentional and Incidental Forgetting in Depression

The central role of memory intrusion as a core feature for the development and maintenance of depression has been pointed out in a recent meta-analysis. Adults with depression are as likely to experience intrusive memories as adults with PTSD [85].

Although memory intrusion appears as a common feature of both PTSD and depression, differences in the mechanisms through which unwanted memories are inhibited emerge from studies assessing intentional forgetting.

While the DF effect seems to be largely unaffected by PTSD, depressed patients submitted to the DF paradigm recalled a higher number of to-be-forgotten words than healthy controls, especially when words were negative and/or illness-related [86,87]. It is important to note that in the studies by Power and collaborators (2000) and Wingenfeld and collaborators (2013), both showing a deficit of DF effect in depression, all the depressed patients met criteria for a diagnosis of Major Depressive Disorder (DSM-IV; APA, 1994). More recently, Xie and collaborators (2018) carried out an event-related potential analysis in healthy subjects with depressive tendencies, assessed through the Beck Depressive Inventory II (average of BDI-II scores was 19 ± 1, which indicates mild depression according to Beck et al., 1996 [88]), during a DF task with neutral and negative words. The study showed that difficulty in suppressing the encoding of negative material in individuals with depressive tendencies correlated with abnormalities in P2 and LPP elicited by word-valence and in P1 and N2 elicited by to-be-forgotten instructions. These results suggested that individuals with early signs of depression could have either an inefficient ability to suppress negative material or an excessive processing of it during encoding [89]. Only one study reported an intact DF effect in patients with a current diagnosis of a depressive episode (average of BDI-II score was 23 ± 1, which indicates moderate depression according to Beck et al., 1996 [88]), but differences in the selection of emotional words among the studies was reported by the authors to account for discrepant results [90].

No differences in the ability to suppress memory retrieval were reported between depressed patients (average of BDI-II score was 31 ± 2, which indicates severe depression according to Beck et al., 1996 [88]) and healthy subjects submitted to a TNT task [91]. However, differences between depressed and non-depressed subjects emerged in brain activity recorded during a TNT task, suggesting that depressed patients use different strategies to inhibit memories when compared to healthy controls [92].

Interestingly, when healthy subjects affected by a mild depression (average of BDI-II score was 19.1 ± 0.6; Beck et al., 1996 [88]) were submitted to a TNT task with neutral words a deficit in suppressing no-think items appeared in these patients. Furthermore, the inability to suppress memory retrieval correlated with the BDI score [19,93]. A mediation analysis revealed that the relationship between the level of depressive symptoms (BDI score) in these subclinical patients and the forgetting rate was fully mediated by working memory capacity [93].

Although speculative, results obtained in depressed patients submitted to intentional forgetting paradigms suggest that deficits in suppressing the retrieval of unwanted memories in TNT could characterize the first stage of depression (namely, in healthy subjects suffering from mild depression). In cases of severe depression, patients learn to suppress retrieval of unwanted memories, although with mechanisms that differ from those used by non-depressed individuals.

In the only study investigating incidental forgetting in depression, Groome and Sterkaj (2010) found that depressed patients, who met DSMIV criteria for the major depressive disorder, submitted to a retrieval practice paradigm for neutral words achieved significantly lower RIF scores than healthy subjects, indicating that depression is associated with a reduced RIF effect [91].

## 4. Intentional and Incidental Forgetting in Schizophrenia

The inability to suppress unwanted information and memory intrusions have been observed in schizophrenic patients, in particular in those with an early onset of the disorder. Recent studies have shown a dysfunctional cognitive control over emotional distraction in patients with schizophrenia [94,95,96,97]. Moreover, an increase in false recognition of distractors in a word recognition task has been found to correlate with the hallucination score [98]. Memory intrusions are also observed in nonclinical individuals with high hallucination scores [99,100], suggesting that deficit in the executive control involved in the inhibition of encoding and retrieval of unwanted memories might be a cognitive marker of the vulnerability to schizophrenia.

In a number of studies testing schizophrenic patients in DF paradigms in which neutral and emotional words were used as stimuli, a deficit in the inhibition of to-be-forgotten items has been observed [101,102,103,104,105,106]. A similar deficit has also been observed in patients affected by Velo-cardio-facial syndrome, a neurogenetic disorder associated with a very high risk for developing schizophrenia [107]. On the contrary, when negative pictures were used as stimuli, schizophrenic patients seemed to display an intact DF effect [108], suggesting that processing of verbal and visual information is differentially affected in schizophrenia.

In studies investigating incidental forgetting through the retrieval practice paradigm for word pairs, an intact RIF effect in the following cued recall test was observed in schizophrenic patients [104,109,110,111].

Together, these results suggest that deficit in intentional, but not in incidental forgetting, could be considered as a cognitive hallmark for the development of schizophrenia.

## 5. Intentional and Incidental Forgetting in Obsessive-Compulsive Disorder

OCD is characterized by recurrent, intrusive, and unwanted thoughts, impulses, and images, often associated with compulsive behaviors that are repetitive, time-consuming, and often ritualized.

Compulsions are generally performed in an attempt to either avoid or neutralize the obsessions and to reduce anxiety. Benzina and collaborators (2016), in reviewing a number of studies on the neuropsychological abnormalities in OCD, revealed an impairment in the decision-making process, behavioral flexibility, verbal and non-verbal episodic memory, inhibitory control, as well as altered attentional processes [112]. Although there is not a broad consensus on the biological and cognitive markers of OCD [112,113,114], the cognitive profile of the disorder seems to be marked by a deficit in executive functions [115,116,117,118]. Chamberlain and collaborators (2005) argued that failures in cognitive and behavioral inhibition could explain clinical symptoms as well as executive deficits observed in memory tasks and in tasks requiring inhibition of prepotent responses [119]. Abnormalities in the cortico-limbic network responsible for the inhibitory control have also been found in OCD patients [120].

Contrasting results emerge when inhibitory control was investigated in OCD patients through the DF paradigm [121,122,123,124,125]. In their pioneering work, Whilelm and collaborators (1996) explored the DF effect in OCD patients in a task in which emotional (both positive and negative) and neutral words had to be forgotten or remembered. Results showed that OCD patients reported significantly more negative to-be-forgotten words than healthy controls in the recall test and in the following recognition task, suggesting that an abnormal encoding of negative information occurred in these patients [121]. Evidence for an abnormal encoding of to-be-intentionally inhibited information based on the emotional valence in OCD was also observed by Bohne and collaborators [122]. Indeed, OCD patients displayed a specific deficit in inhibiting the retrieval of information with negative valence [122].

Tolin and collaborators (2002) extended the lack of DF effect in OCD patients for disease-relevant words, independently of their valence (positive and negative). Interestingly, this lack of DF effect was observed in OCD patients, but not in anxious subjects who displayed a forgetting rate comparable to that of healthy controls [123].

In a more recent study, Moritz and collaborators (2011), by using a DF paradigm in which washing, checking, neutral, and negative words were presented, failed to find a lack of DF effect in OCD patients. However, they reported an unusual counter effect, according to which to-be-forgotten words referring to washing and checking were better remembered than to-be-remembered words in healthy controls [124], suggesting a bias in stimuli selection. In the same year, Konishi and collaborators (2011) found a normal DF effect in OCD for neutral words. Despite an intact DF effect, OCD patients appeared to recall fewer to-be-remembered words than healthy controls [125].

As concerns incidental forgetting, Jelinik and collaborators (2012) found an intact RIF effect in OCD patients submitted to a retrieval practice paradigm, in which OCD-relevant, neutral, and negative words were used as stimuli to be encoded. However, when the RIF effect was specifically analyzed for salient OCD-relevant words, a slight deficit was observed in patients [126].

A lack of RIF effect was reported in OCD patients suffering from mild depression submitted to a retrieval practice task in which neutral words were used as stimuli [116].

Together the above reported studies seem to suggest that OCD patients are unable to intentionally forget negative disease-relevant stimuli.

## 6. Neurotransmitters and Active Forgetting

Several neurotransmitter systems are known to be involved in the acquisition, consolidation, and retrieval of memories. In recent years, neurobiologists discovered neural, cellular, and molecular processes actively involved in erasing the substrate of memory or in suppressing its accessibility [22,23]. In this section we review studies concerning the involvement of neurotransmitters in active forgetting by underlining, where possible, their role in incidental and intentional forgetting.

### 6.1. Glutamate

Glutamate is recognized as the major excitatory neurotransmitter in the mammalian brain and exerts its excitatory function through both ionotropic and metabotropic receptors. NMDA, AMPA, and kainate are inotropic receptors, while mGluRs are a class of metabotropic receptors [127].

Among glutamate receptors, NMDA receptors are largely known to be involved in learning and memory processes, as well as in long-term potentiation. Pre- or post-training administration of either competitive (e.g., AP5) or non-competitive (e.g., MK-801, ketamine) NMDA antagonists prevents acquisition and impairs memory consolidation in different animal species submitted to different tasks [128]. Moreover, there is evidence about the involvement of NMDA receptors in acquisition and retention of extinction [129,130], in long-term depotentiation [131], as well as in spontaneous forgetting [132]. In particular, Quartermain and collaborators (1991) found that the administration of milacemide, a metabolic precursor of glycine, which in turn potentiates NMDA activity, suppressed the time-dependent decay of memory for both active and passive avoidance tasks [132]. In a recent work, Sachser and collaborators (2016) found that pharmacological inhibition of NMDA receptors, through systemic administration of memantine and MK801, prevented the normal forgetting of object-location memory in rats, as well as long-term potentiation (LTP) decay driven by the GluN2B subunit of NMDA receptor. In addition, they found that the time-dependent memory loss required Ca^2+^ influx. Systemic administration of L-type voltage-dependent Ca^2+^ channel (LVDCC) blocker (nimodipine) was, in fact, effective in maintaining long-term memory for object location. Similarly, the inhibition of the calcium-dependent phosphatase calcineurin (CaN) prevented memory loss [133]. Interestingly, CaN activity through NMDA and LVDCC are involved in AMPA trafficking, it is thus possible to hypothesize that the removal of AMPA receptors from the membrane through the influx of calcium after memory consolidation induced forgetting [134].

Blocking the synaptic removal of the GluA2 subunit of AMPA receptors prevents the natural time-dependent forgetting of long-term memories [135,136].

More recently, a differential involvement of GluN2A and GluN2B subunits of the NMDA receptors on memory retention and forgetting has been observed [137]. The intra-hippocampal administration of the GluN2A antagonist, but not the GluN2B antagonist, after the acquisition of a Morris water maze task suppressed spatial memory decay in rats. Conversely, the GluN2B antagonist, but not the GluN2A antagonist, downregulated memory retention in the following memory test [137].

Together these results suggest the active role of the glutamatergic system in forgetting.

### 6.2. GABA

The GABAergic system is the main inhibitory system in the mammalian central nervous system [138]. It is known to influence neuronal development and synaptic plasticity, as well as learning and memory processes [139,140]. Two receptor systems bind GABA: GABA_A_ and GABA_B_ receptors.

GABA_A_-ionotropic receptors are particularly abundant in the amygdala [141], where they play a major role in the neural mechanism underlying inhibition of aversive memory, such as fear extinction [142], while GABA_B_-metabotropic receptors are necessary to control both short- and long-term memory formation [143].

In infant rats (16-, 18-day-old) submitted to a fear conditioning task, and tested 48 h after training, a pre-test administration of a GABA_A_ inverse agonist prevented the long-term memory forgetting typically observed in developing rats. The latter suggests a role for GABA_A_ in infantile amnesia [144,145,146].

In a single case human study, chronic intrathecal administration of the GABA_B_ agonist baclofen induced a transient amnesic syndrome, associated with accelerated forgetting for autobiographical memories, suggesting a role for GABA_B_ in modulating time-dependent forgetting [147].

### 6.3. Acetylcholine

The cholinergic system in the brain encompasses two major pathways: (i) the basal forebrain cholinergic system, including the nucleus basalis of Meynert (NBM), the medial septal nucleus, and the diagonal band of Broca (MSDB). The basal forebrain cholinergic system has extensive projections to neocortical regions, as well as to basolateral amygdala and olfactory bulb, hippocampus, and entorhinal cortex. (ii) The brainstem cholinergic system, including the pedunculopontine nucleus and the laterodorsal pontine tegmental nucleus. This brainstem system projects primarily to thalamic structures and to basal forebrain regions.

Acetylcholine released by presynaptic neurons exerts its effect on post-synaptic neurons by binding ionotropic nicotine receptors and metabotropic muscarine receptors. The activation of both nicotine- and muscarinic-cholinergic receptors is involved in either acquisition or extinction learning, indicating that cholinergic regulation plays a fundamental role in memory formation and consolidation [148,149]. In particular, the basal forebrain projections to prefrontal cortex, hippocampus, amygdala, parietal, and sensory regions are known to be involved in learning and memory processes, as well as in extinction of conditioned memories [150,151]. In the hippocampus, nicotine receptors are particularly localized postsynaptically on GABAergic neurons and play a major role in regulating excitatory neurotransmission. Instead, muscarinic receptors are mainly localized on hippocampal glutamatergic pyramidal neurons and are believed to provide direct excitatory input from basal forebrain afferents, strengthening synaptic plasticity in hippocampal networks [151]. Several emerging lines of evidence suggest that cholinergic modulation of the cortico-hippocampal-amygdala circuit may regulate specific aspects of learning and forgetting [148].

However, contrasting results emerge when the role of cholinergic system in active and passive forgetting are specifically considered.

Edgiton and Rusted (2003) found that an acute dose of nicotine (obtained by smoking a cigarette from the preferred brand), in smokers who were asked to abstain from smoking 2 h before test, increased RIF effect without affecting memory recall. Conversely, in the same subjects the acute administration of scopolamine (a muscarinic cholinergic receptor antagonist), 1 h before training, impaired memory recall, but did not affect RIF [152]. These results suggest that the cholinergic receptor system is differentially involved in memory formation and forgetting, indicating a pivotal role for nicotine receptors in modulating incidental forgetting through inhibitory control. More recently Rusted and Alvares (2008) found that the administration of a single dose of nicotine (obtained by a nasal spray administration) in non-smoker participants enhanced the RIF effect. Interestingly, this enhancement was not observed in subjects submitted to a non-pharmacological procedure aimed at enhancing, like nicotine, the arousal level. These results lend some support for a specific role of nicotine receptors in the inhibitory control mechanism involved in incidental forgetting [153].

### 6.4. Dopamine

Dopaminergic neurons in the brain largely originate from the substantia nigra (SN) pars compacta and ventral tegmental area (VTA), forming the nigrostriatal and mesocorticolimbic pathways, respectively. In humans, midbrain dopaminergic neurons from VTA project to the prefrontal cortex (PFC) via the mesocortical pathway and to the nucleus accumbens, hippocampus, and amygdala via the mesolimbic pathway. This mesocorticolimbic system plays a role in reward, motivation, arousal, learning, and memory [154].

Several studies on the dopaminergic system have shown that dopamine release facilitates memory consolidation in different species submitted to both appetitive and aversive learning paradigms [155,156,157,158]. Interfering immediately after learning with dopamine activity impaired memory consolidation in drosophila, mice, and humans [22,159,160,161,162].

Although memory loss due to an impairment in dopamine signaling indicates its involvement in passive forgetting, recent studies seem to demonstrate that the ongoing dopamine activity after learning contributes to memory erasure, suggesting a dopamine-dependent active forgetting [163,164,165,166].

In an elegant study on drosophila, Berry and collaborators (2012) found that two different subsets of dopaminergic neurons are involved in retention and forgetting of both aversive and rewarding memories [159]. Blocking the output of dopamine-forgetting neurons after learning resulted in an enhancement of memory expression, while the stimulation of these neurons accelerated memory decay, indicating that dopamine is involved in active forgetting and that memory consolidation may counter the activity of dopamine-forgetting neurons [159]. Post-training stimulation of dopamine-forgetting neurons in drosophila, through locomotor activity, promotes the forgetting of aversive olfactory memories, while the inhibition of these neurons, through post training administration of the GABA-A agonist Gaboxadol, counteracts forgetting by facilitating memory consolidation [164]. These results suggest that the ongoing activity of dopaminergic neurons could determine the memory outcome: a strong dopaminergic activity immediately after acquisition determines forgetting, while its reduction facilitates memory consolidation.

Consistently, the administration of the monoamine stabilizer (-)-OSU6162 blocks the delay-dependent forgetting of object location memory in mice [167]. Interestingly, the (-)-OSU6162 acts as a dopaminergic stabilizer, by either inhibiting or stimulating the dopaminergic transmission depending on the dopaminergic tone. Hence, it is possible to hypothesize that counteracting aberrations in dopaminergic signaling, without interfering with its normal functioning, might prevent forgetting.

In humans, chronic and recreational cocaine users showed a reduction in the DF effect, indicating that abnormal dopaminergic transmission interferes with the ability to intentionally suppress unwanted memories [167]. Interestingly, a recent neurogenic study demonstrated that in adulthood the presence of one or more polymorphisms associated with higher DA signaling predicted the forgetting rate in a picture recognition task [168]. Furthermore, humans with the Met/Met polymorphism in the catechol-O-methyltransferase gene (which leads to higher prefrontal dopamine availability) displayed a greater RIF effect, when compared with Val/Met and Val/Val allele carriers [169].

Together, these results support the hypothesis that the cortical dopaminergic system is centrally involved in the inhibitory control of memory, determining intentional as well as incidental forgetting.

### 6.5. Noradrenaline

Noradrenaline is an important hormone and neurotransmitter majorly secreted from the locus coeruleus located in the pons of brain stem. Noradrenergic projections from the locus coeruleus (LC) are directed to (i) the spinal cord and abdomen, where this neurotransmitter is used in sympathetic ganglia; (ii) the neocortex, including PFC; and (iii) the limbic system. The noradrenergic system is involved in multiple complex behavioral regulations, such as the regulation of arousal levels and vigilance [170].

There is evidence indicating that noradrenaline modulates memory formation for emotionally salient events [171]. Amygdala activation during the encoding of emotionally arousing stimuli has been shown to depend on the noradrenergic system [172,173,174,175], and pharmacological manipulations that increase the central release of noradrenaline in response to emotional arousal improve episodic memory formation [176,177,178]. In contrast, the administration of the adrenergic receptor antagonist propranolol blocks this emotional enhancing effect [174,175].

As concerns the role of the noradrenergic system in determining remembering or forgetting, interesting results emerge when the emotional valence of to-be-learned items is considered. Hurlemann and Collaborators (2005) found that the presence of negative material impaired the subsequent recall of episodic memories (retrograde amnesia), while the presence of positive material determined a better recall (retrograde hypermnesia). The pharmacological modulation of the noradrenergic system—blocking or enhancing noradrenergic transmission by the administration of either beta-receptor antagonist or noradrenalin reuptake inhibitor, respectively—was able to modify the levels of amnesia or hypermnesia, suggesting that memory persistence or forgetting was a function of the emotional arousal due to noradrenergic signaling [179]. In rats, the pre-test administration of either beta or alpha noradrenergic agonists has been observed to alleviate the time-dependent forgetting of an active avoidance memory [180]. Conversely, noradrenaline-depleted mice submitted to a water maze task displayed a more rapid forgetting than control mice [181]. Together these results seem to support the idea that noradrenaline-mediated arousal could play a role in determining which information has to be remembered or forgotten.

### 6.6. Glucocorticoids

It is well established that stress affects memory consolidation and reconsolidation through glucocorticoid release [182,183,184]. The hypothalamus–pituitary–adrenal (HPA) axis leads to the release of glucocorticoid hormones from the adrenal cortex under stress conditions. Glucocorticoids enter the blood–brain barrier and bind glucocorticoid (GR) and mineralocorticoid receptors MR. GRs are highly ubiquitous and expressed in most brain regions (including PFC), whereas MRs are predominantly expressed in limbic regions such as the hippocampus and amygdala [185]. Through this binding with GR and MR, glucocorticoids act on memory formation enhancing consolidation and impairing retrieval [186,187].

Although there is a general consensus on the role of glucocorticoids on memory formation, few studies have systematically investigated their role in memory forgetting.

Koessler and collaborators (2009; 2013) found that oral administration of cortisol did not affect RIF, although stress-induced increases in salivary cortisol levels were able to eliminate the RIF effect [188,189].

Similarly, subjects submitted to a stress-induced procedure, able to increase cortisol levels, showed an impairment in the ability to intentionally inhibit retrieval of memory in a TNT paradigm. This deficit in memory control was related to the stress-dependent modulation of functional connectivity between the hippocampus and prefrontal cortex linked to memory suppression [190].

In a recent study, Kuehl and collaborators (2017) showed that acute cortisol administration before a DF task, in which participants were instructed to remember or to forget emotional words, did not affect memory performance [90].

Overall, these results seem to suggest that cortisol released due to stress induction plays a role in both incidental and intentional forgetting.

### 6.7. Neurotransmitters, Inhibitory Control, and Active Forgetting: An Overview

Several lines of evidence from brain imaging studies, as well as findings from psychiatric and neurological patients, indicate the main role of prefrontal cortex in orchestrating the inhibitory control of response behavior, emotions, as well as the retrieval of unwanted memories. The neural mechanisms and neurotransmitters involved in these PFC functions are extensively described elsewhere in excellent reviews [191,192,193,194,195,196,197,198,199].

In this paragraph we tentatively summarize the possible role of the neurotransmitter systems described above in the neural mechanisms involved in the inhibitory control of memories that may lead to active forgetting.

**Figure 2:**
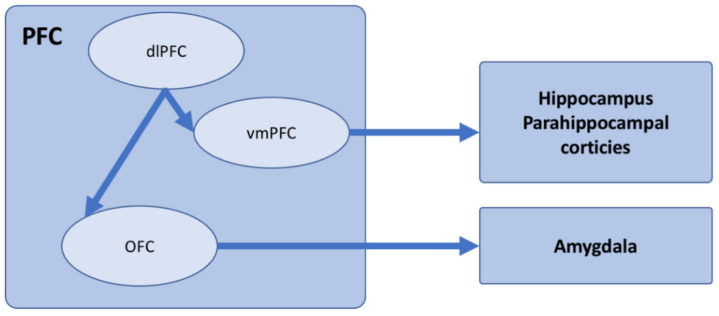
Schematic showing the neural network driving inhibitory control of unwanted memories.

Anderson and colleagues (2016; 2020), by extensively reviewing several works on the neural networks involved in the inhibitory control of memories, reported that the prefrontal cortex (PFC) inhibits the activity of subcortical structures (e.g., the hippocampus) during RIF, TNT, and DF [191,196,200]. In particular, a PFC network—which includes connections between dorso-lateral PFC regions (dlPFC) with medial PFC (including the anterior cingulate cortex, aCC), and with the orbitofrontal cortex (OFC)—influences the activity of hippocampus, parahippocampal cortices, and amygdala (see Figure 2). The inhibitory control exerted by PFC on the hippocampus during memory suppression has been extensively demonstrated in functional activation and connectivity studies. An inhibitory control of PFC on the parahippocampal cortices and amygdala has been further observed when memories for a scene’s spatial context and for emotional content had to be suppressed, respectively [196,200].

**Figure 3:**
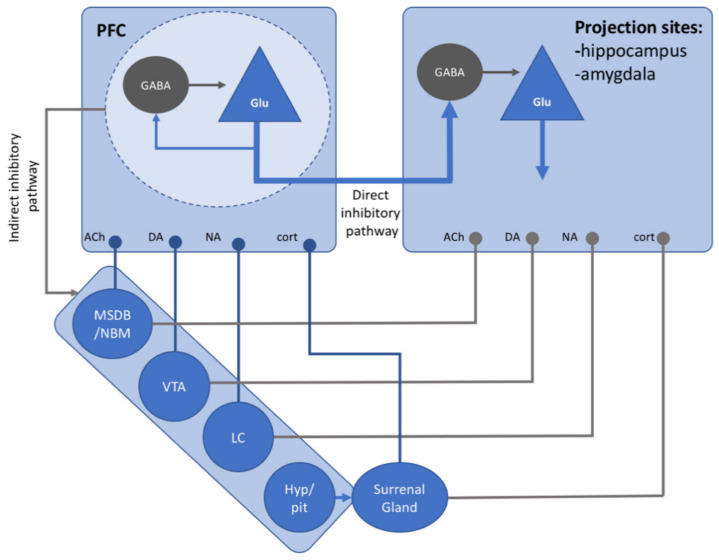
Schematic showing the cellular mechanism of direct and indirect inhibition driven by glutamatergic neurons in the prefrontal cortex (PFC). Afferent pathways from the main neurotransmitter systems, known to modulate PFC activity in inhibitory control, are also depicted.

At the cellular level, PFC function is related to the activity of pyramidal glutamatergic neurons and GABA interneurons. Efferent pathways from PFC are mainly due to glutamatergic projections toward cortical and subcortical structures. The activity of glutamatergic neurons is finely tuned by local inhibitory GABAergic interneurons. This cellular network, which consist of pyramidal glutamatergic neurons and GABA interneurons, may be involved in the enhanced processing of relevant stimuli as well as in the inhibition of irrelevant stimuli, thereby facilitating memory formation or forgetting. The inhibitory activity of PFC on cortical and subcortical structures seems to be driven by the firing of excitatory glutamatergic neurons, which directly excite local inhibitory GABAergic neurons located in the subcortical sites (e.g., hippocampus) responsible for memory formation (“direct inhibition”) (Figure 3).

The PFC control over hippocampus and amygdala can also follow an “indirect” pathway. PFC can indirectly modulate the activity of the hippocampus and amygdala through glutamatergic projections to the ventral tegmental area (VTA), locus coeruleus (LC), and basal forebrain, which in turn send dopamine, noradrenaline, and acetylcholine axons to both the hippocampus and amygdala [201,202]. The “direct inhibition” exerted by PFC glutamatergic projections to GABAergic neurons of subcortical structures like the hippocampus and amygdala appears to be the main pathway through which memories are suppressed [203]. Recently, imaging results obtained in humans submitted to a memory suppression task have revealed that higher concentrations of hippocampal GABA correlated with a greater downregulation during retrieval suppression and greater negative coupling between the PFC and the hippocampus, supporting the idea of a direct inhibitory PFC–hippocampus pathway [204].

Similarly, Depue and collaborators (2007) found that PFC directly inhibits the activity of the hippocampus and amygdala during memory suppression by using an emotional version of the TNT task [205]. Furthermore, direct projections from the PFC to GABAergic inhibitory neurons, located in intercalated cell masses of the amygdala, have been extensively observed in non-human primates and rodents [203,206,207,208].

Acetylcholine, dopamine, noradrenaline, and glucocorticoid are known to modulate the activity of glutamatergic and GABAergic neurons in the PFC and to shape the dendritic arborization of these neurons [209,210,211,212,213].

Cholinergic neurotransmission in the neocortex is known to be involved in several cognitive functions that include perception, attention, emotion, and memory consolidation [130,213,214]. Recently, a role for cholinergic transmission in the inhibitory control has also been hypothesized. In a recent fMRI study, Kasparbauer and colleagues (2018) found that the dlPFC is activated during the execution of a response inhibition task. Interestingly, nicotine administration increased dlPFC activity when response inhibition was successfully exercised. The magnitude of dlPFC activity due to nicotine administration was related to trait impulsivity: the higher the level of impulsivity, the stronger the reduction in dlPFC activity induced by nicotine when subjects failed to correctly inhibit the response [215]. As previously discussed in Section 6.1, nicotine administration in non-smoker subjects increased the RIF effect. Hence, although speculative, these results seem to indicate that cholinergic transmission, mediated by nicotine receptors in PFC, is essential for modulating the inhibitory control required for active forgetting.

Noradrenergic projections from LC and dopaminergic projections from VTA are classically associated with arousal regulation [216,217,218,219,220]. Release of noradrenaline and dopamine in PFC is low during sleep, moderate during alert situations, and high during uncontrollable stress [204]. Recent findings support the notion that the arousal-related release of both dopamine and noradrenaline into the PFC is involved in gain control mechanisms, which in turn amplify task-relevant signals by reducing neuronal noise. Such a gain control seems to be strongly involved in the inhibitory control process driven by PFC [204,221,222,223,224]. Pharmacological and imaging studies revealed that increasing dopaminergic and noradrenergic transmission (by the administration of psychostimulants, the dopamine/norepinephrine transporter blocker methylphenidate, or the noradrenaline inhibitor reuptake atomoxetine) are able to increase response inhibition. Such an increase in response inhibition correlated with the magnitude of PFC activation [196,225,226,227,228,229,230,231]. Both noradrenaline and dopamine are potent neuromodulators involved in the regulation of remembering and forgetting. In particular, the level of dopamine during the original encoding experience seems to determine whether memories are forgotten or remembered. Similarly, noradrenaline levels mediated by arousal could play a role in determining which information has to be remembered or forgotten. Hence, by extending results on response inhibition to memory control, it is possible to envisage that the inhibitory control of memories mediated by the PFC is under the influence of dopaminergic and noradrenergic tone. Interestingly, both dopamine and noradrenaline have a U-inverted effect on performance mediated by the PFC function, such that both the lowest and the highest levels of dopamine or noradrenaline impair performance [232].

In the last decades, neuroendocrine research has indicated that glucocorticoids modulate behavioral response and cognitive performance in stress conditions, particularly hippocampus-mediated declarative memory and prefrontal cortex-mediated working memory for emotional material [184,185,186,233].

Acute stress appears to dampen input onto inhibitory neurons, which results in an increase in glutamate release of PFC neurons. Such a glutamate release depends on glucocorticoid-related mechanisms [199]. Chronic stress also affects PFC function by remodeling the neural architecture of PFC (dendritic shrinking mPFC and dendritic expansion in OFC) [197]. Recent experimental results show that stress-dependent cortisol release impairs the inhibitory control of memories as well as response inhibition in a go/no-go task [174,190,234].

Taken together, results discussed in this section suggest that (i) overlapping PFC networks are involved in behavioral (e.g., stopping a motor response) and cognitive (e.g., memory suppression) inhibition; (ii) active forgetting is due to the inhibitory control exerted by PFC on subcortical structures (i.e., hippocampus, amygdala, and parahippocampal cortices) responsible for memory formation; and (iii) different neurotransmitter systems modulate the function of PFC networks responsible for inhibitory control.

## 7. Conclusions

In summary, the results of the studies discussed in this review suggest that deficits in the inhibitory control involved in forgetting of unwanted memories are a common feature of psychopathologies like PTSD, depression, schizophrenia, and OCD. Furthermore, alterations in PFC–subcortical networks responsible for the inhibitory control mechanisms involved in active forgetting have been documented in schizophrenia, depression, OCD, and PTSD [210,231,235,236]. When incidental and intentional forgetting are investigated in patients, different patterns of deficits have been observed among these psychopathologies (see Table 1).

Moreover, when pharmaco-behavioral evidence on the role of neurotransmitter systems in the inhibitory control of memories is put in relation to the deficits observed in patients, an interesting pattern of results emerges from the analysis of the literature.

As reported in Section 6.7, glutamatergic pyramidal neurons and GABAergic local interneuron in PFC play a pivotal role in driving the inhibitory control of memories. Alterations in glutamatergic and GABAergic transmissions have been reported in schizophrenia, depression, PTSD, and obsessive-compulsive disorder [237,238,239,240,241,242,243]. Moreover, studies on the role of these neurotransmitter systems in mental disorders seem to be promising for better understanding etiopathogenetic mechanisms, as well as for improving the effectiveness of pharmacological therapies [244,245,246,247,248,249,250]. Although direct evidence on the role of glutamatergic and GABAergic transmissions in active memory forgetting in psychopathological disorders is still lacking, it is possible to hypothesize that dysfunctions of these neurotransmitter systems are a common basis for deficits in active forgetting observed in the different psychopathologies.

Specific dysfunctions in other neurotransmitter systems, known to modulate the activity of glutamatergic and GABAergic neurons in PFC, might account for the different patterns of deficits that have been observed in active memory forgetting among psychopathologies.

Severe deficits in intentional inhibition of memory retrieval in the TNT paradigm and slight deficits in the incidental inhibition of memory retrieval in the RIF paradigm are observed in PTSD patients. On the other hand, intentional inhibition of memory encoding in DF tasks seems to be largely spared in these patients. On the contrary, a lack of both DF and RIF effects, but not TNT, are observed in depressed patients.

Studies in which intentional and incidental forgetting have been investigated in relation to cortisol release induced by stressful events seem to parallel the deficits in the inhibitory control of memory retrieval observed in PTSD patients. In particular, stress-induced cortisol release impaired RIF and TNT, but not DF effect [184,186,251]. A reduction in the ability to suppress the retrieval of unwanted memories related to an increase in cortisol level has been reported in PTSD patients. It is thus possible to hypothesize that post-traumatic stress might affect the inhibition of traumatic memories through alterations in cortisol release. Conversely, it is possible to envisage that individual differences in the capability to inhibit the retrieval of unwanted memories might be a risk factor for the development of PTSD after trauma exposure. In healthy subjects, the ability to inhibit unwanted memories negatively correlated with the presence of intrusions by a previously watched traumatic movie [252].

Imaging studies show that emotion dysregulation in PTSD patients is related to a reduced PFC volume and to a weak inhibitory connection between the PFC and amygdala [253]. Furthermore, imaging and lesion studies in animal models of PTSD provide further support to the possible role played by the stress-induced deficits in the inhibitory control of memories due to a breakdown of PFC–amygdala connectivity [252,253].

Alterations in cortisol release, as well as reduced inhibitory control driven by PFC, are also observed in depressed patients [254,255]. PTSD and depression comorbidity due to exposure to traumatic experiences has been reported [256]. Hence, dysregulation in cortisol release due to traumatic stress may account for deficit in active forgetting observed in both PTSD and depressed patients.

The double dissociation observed in intentional forgetting between PTSD and depression might be explained by indirect pieces of evidence. As aforementioned, PTSD patients show an intact DF effect but a reduced TNT effect, while depressed patients display a strong reduction in DF effect, but a normal TNT. At the neurobiological level, a reduction in dopamine release has been strongly associated with depressive symptoms [257], while an increase in noradrenergic transmission following a traumatic event is correlated with PTSD symptoms [258]. Alterations in dopaminergic, but not noradrenergic, transmission seem to be involved in DF. It is thus possible to envisage that differences between PTSD and depression in the level of catecholamine release may account for the behavioral differences observed in patients submitted to the intentional forgetting paradigms.

Furthermore, recent clinical studies have pointed out a role for the cholinergic system in depression. In particular, reduced activity of nicotine receptors has been related to the onset of depression-like behaviors, while nicotine administration improved mood in depressed patients two days after its administration [259]. Pharmacological studies demonstrated a specific role for nicotine acetylcholine receptors in incidental forgetting, investigated through the RIF paradigm [260,261,262]. It is worth noting that, at the cognitive level, nicotine administration increases the RIF effect, which is impaired in depression, suggesting a close relationship between cholinergic alteration and incidental forgetting deficit in depression.

A specific deficit in DF emerges in studies in which memory forgetting has been investigated in schizophrenic patients. Such patients, differently from depressed patients, showed an intact RIF effect. The fact that a reduction in the RIF effect has been reported in depression, but not in schizophrenia, suggests that assessing the ability to involuntary inhibit unwanted memory could be a useful tool to differentiate diagnosis in these patients, especially at the early onset of these psychopathologies [106,251,263,264,265].

Although speculative, it is possible to envisage that alterations in dopamine activity (namely, reduced dopamine levels in depression and enhanced in schizophrenia), together with an altered capability to inhibit the encoding of unwanted memories, could be considered as hallmarks of schizophrenia and depression. Interestingly, dopamine levels affect PFC-dependent processes in a U-inverted fashion: the highest and lowest levels of dopamine impair performance [233].

As in depressed and schizophrenic patients, OCD patients showed a lack of DF effect, especially when OCD-related stimuli have to be intentionally suppressed, while RIF is only slightly affected. Interestingly, a severe deficit in RIF effect in OCD patients has been observed in those subjects suffering from depressive symptoms. These pieces of evidence suggest that deficits in the mechanisms driving incidental forgetting are specifically compromised by depressive states.

OCD patients display a profound inability to inhibit intrusive thoughts and compulsive behaviors. Dysfunctions of the PFC network involved the inhibitory control process, perhaps due to altered dopaminergic transmission, might also help to explain comorbidities between OCD and other psychopathologies such as depression and schizophrenia [112,119,195,266,267].

The above reported results are intriguing for the definition of possible biological and cognitive hallmarks for the development of psychopathologies like PTSD, depression, schizophrenia, and OCD.

There is growing support for the notion that cognitive control abnormalities are a central component of many of the neuropsychological deficits observed in individuals with mental illnesses. Cognitive control refers to a set of mental processes that modulate other cognitive and emotional systems in service of goal-directed adaptive behavior [268]. Among the executive functions related to the cognitive control, the inhibition of unwanted memories through active forgetting emerges as a critical mechanism in order to maintain mental health. Deficits in this function have been observed in several psychopathologies [23,191].

A number of limits in the studies reviewed have to be carefully taken into account before the role of active forgetting can be considered in the assessment of psychopathologies.

First, the mechanisms of forgetting in patients were investigated in studies with small sample sizes; thus, the generalizability of results could be compromised. As reported in a recent review, sample size is one of the critical factors in considering putative bio-behavioral markers of psychopathologies as possible endophenotypes [269]. A second limit regards the ecological validity of stimuli used to investigate suppression of unwanted memories. Most studies used (emotional and/or disorder-related) words. It is possible to hypothesize that different results would be obtained with more ecologically valid stimuli. For example, there is evidence showing that emotional movies are better than words or pictures to investigate emotional memory process in PTSD [270]. Third, all the patients involved in the studies underwent medical treatments that per se could affect the inhibitory mechanisms involved in forgetting. It is thus possible that different patterns of results on forgetting could emerge in unmedicated patients or in real-life conditions. For this reason, studies on the involvement of active forgetting in psychopathology could benefit from future investigations in unmedicated healthy subjects, at risk to develop a mental disorder, or at the early stages of the psychopathological development.

Overall, although preliminary, the results we discussed in this review seem to provide pieces of evidence in favor of the use of active forgetting paradigms to increase pre-clinical tools aimed at the identification of the early onset of psychopathology and to aid clinicians in the therapeutic decision-making process.

## Figures and Tables

**Table 1:** Intentional and incidental forgetting affected by psychopathologies.

	Intentional Forgetting		Incidental Forgetting
	DF	TNT	RIF
Post-traumatic stress disorder	unaffected	affected	slightly affected
Depression *	affected	unaffected	affected
Schizophrenia	affected	n.a.	unaffected
Obsessive compulsive disorder	affected (only for disease-related stimuli)	n.a.	slightly affected (affected in OCD patients suffering of depressive symptoms)

DF: direct-forgetting; TNT: think/no-think; RIF: retrieval-induced forgetting. * Includes patients with a diagnosis of Major depressive disorder and subjects with mild to moderate depression.
